# Mechanical Properties of Surface-Charged Poly(Methyl Methacrylate) as Denture Resins

**DOI:** 10.1155/2009/841431

**Published:** 2009-04-12

**Authors:** Sang E. Park, Maggie Chao, P. A. Raj

**Affiliations:** ^1^Department of Restorative Dentistry and Biomaterials Sciences, Harvard School of Dental Medicine, 188 Longwood Avenue, Boston, MA 02115, USA; ^2^Private practice, 1111 Civic Drive, Suite 321, Walnut Creek, CA 94596, USA; ^3^Division of Research and Development, Perident Therapeutics, Inc., 5611 West Morgan Avenue, Unit L, Milwaukee, WI 53220, USA

## Abstract

The aim of this study was to examine the mechanical properties of a new surface-modified denture resin for its suitability as denture base material. This experimental resin is made by copolymerization of methacrylic acid (MA) to poly(methyl methacrylate) (PMMA) to produce a negative charge. Four experimental groups consisted of Orthodontic Dental Resin (DENTSPLY Caulk) as a control and three groups of modified PMMA (*m*PMMA) produced at differing ratios of methacrylic acid (5 : 95, 10 : 90, and 20 : 80 MA : MMA). A 3-point flexural test using the Instron Universal Testing Machine (Instron Corp.) measured force-deflection curves and a complete stress versus strain history to calculate the transverse strength, transverse deflection, flexural strength, and modulus of elasticity. Analysis of Variance and Scheffe Post-test were performed on the data. Resins with increased methacrylic acid content exhibited lower strength values for the measured physical properties. The most significant decrease occurred as the methacrylic acid content was increased to 20% *m*PMMA. No significant differences at *P* < .05 were found in all parameters tested between the Control and 5% *m*PMMA.

## 1. Introduction

Denture stomatitis is a common form of oral Candidiasis, which is associated with the adherence of *Candida albicans* to denture base surfaces [1–4]. *Candida* is a commensal organism that is frequently present in healthy individuals. Introduction of predisposing factors such as systemic disease, immunosuppressive drugs, xerostomia, or dentures result in fungal infections [5, 6]. Candidiasis has been associated with increased numbers of *Candida albicans* particularly on the tissue-fitting surface of maxillary complete dentures. Maxillary denture wearers are more susceptible to *Candida* infections since the denture base serves as an effective reservoir harboring microorganisms. Low salivary flow rates, low buffering capacities, and low pH values under dentures contribute to colonization of the oral mucosa and denture surfaces by *Candida* [7–12].

Development of pathogenesis is preceded by the initial attachment of *Candida* on the palatal mucosa and mucosal surface of the denture. Surface characteristics resulting from chemistry are significant in the initial adherence of *Candida* to the denture resin and offer an opportunity for further bonding and colonization [13–15]. *C. albicans* has a net negative surface charge, providing an environment of electrostatic repulsion through the negative-negative charge interactions with the polymer. Understanding the effect of electrostatic interaction in the adhesion of *C. albicans* to poly(methyl methacrylate) (PMMA), our previous research supported the hypothesis that negatively charged denture base materials can prevent adhesion of *C. albicans* and reduce the development of denture-induced stomatitis [16].

Poly(methyl methacrylate) (PMMA) is the resin of choice for fabrication of denture bases in clinical dentistry. It has excellent physical properties and clearly defined polymerization process that is easy for modification. Many attempts have been made to modify PMMA taking advantage of the broad scope of modification available in polymer chemistry. In our previous study [16], the experimental resin had a negative charge incorporated by copolymerization of methacrylic acid to methyl methacrylate. Results showed that the adhesion of *C. albicans* decreased significantly as the ratio of methacrylic acid increased in vitro. A significant decrease in Candidal adhesion to the resin samples (*P* < .0001) existed when the methacrylic acid was present at 10% of the modified PMMA. These positive findings made the new surface-modified denture resins attractive for future dental applications.

An optimized resin material should exhibit a positive biologic response (i.e., decreased adhesion of *Candida*) while maintaining the desired physical properties. Physical and mechanical properties of polymers are crucial in achieving clinical success and longevity of complete dentures fabricated. Important physical properties include the following: compressive and tensile strengths; elongation; hardness; thermal characteristics; molding properties; polymerization shrinkage; solubility; dimensional stability; and dimensional accuracy [17]. One of the most critical characteristics of a denture base resin is strength. The denture base must be able to withstand high impact forces in addition to normal masticatory forces.

Denture base fractures have been examined using different testing protocols. Strength testing can include compressive, shear, tensile, transverse, impact, and fatigue strength. Our earlier study exhibited some physical properties that warrant further investigation. Microcracks were observed under light microscope, especially in modified PMMA samples that had higher methacrylic acid content suggesting that increasing the ratio of methacrylic acid may compromise the physical properties of the resin [16]. In light of these findings, it is important to elucidate the physical properties of these surface-modified resins. 

The aim of this study was to investigate the mechanical properties of a new surface-modified PMMA in terms of transverse strength, transverse deflection, flexural strength, and modulus of elasticity for its application as denture base resin.

## 2. Materials and Methods

### 2.1. Synthesis of Modified PMMA Polymers

Modified PMMA polymers were synthesized by polymerization of mixtures of varying proportions of methyl methacrylate (MMA) and methacrylic acid (MA) as monomers. They included 5% *m*PMMA (5% MA and 95% MMA), 10% *m*PMMA (10% MA and 90% MMA), and 20% *m*PMMA (20% MA and 80% MMA). In a 50 mL flask, 35 g of the monomer or the monomer mixture was stirred with 1.2 g of benzoyl peroxide. Subsequently, 0.75 mL of dimethyl paratoluidine was added and stirred briefly. The mixture was poured into a 250 mL flask containing 1% poly (vinyl alcohol) at pH 3 and stirred well to prevent separation of two layers, and the temperature was recorded. The reaction was allowed to continue for 15 minutes after the rise in temperature ceased. The polymer beads were filtered, washed with distilled water, and dried. All chemicals were obtained from Sigma-Aldrich Chemical Co., Inc., Milwaukee, WI.

### 2.2. Characterization of Modified PMMA Polymers

The synthesized PMMA and modified PMMA polymers were analyzed by FTIR (Model FTS-40, BioRad laboratories, Richmond, CA) for the incorporation of carboxylate group. The changes in the region 2800–3100 cm^−1^ in the FTIR spectra of carboxylated polymers showed significant broadening of bands between 2950–3050 cm^−1^ as compared to that of PMMA. In addition, the appearance of new IR bands at 2924 and 2885 cm^−1^ for the modified PMMA polymers suggested the incorporation of carboxylate group.

### 2.3. Preparation of Resin Samples

Three groups of modified PMMA (5% * m*PMMA, 10% * m*PMMA, 20% * m*PMMA) and one commercially available dental resin, Orthodontic Dental Resin (DENTSPLY Caulk, Milford, DE), were included in the study. The experimental groups were designated as the following: Group 1(Control)-Dental Resin; Group 2–5% *m*PMMA; Group 3–10% *m*PMMA; Group 4–20% *m*PMMA. Orthodontic Dental Resin was fabricated according to manufacturer's instructions. Resin samples in Groups 2–4 were polymerized using chemicals in the ratios shown in Table 1 (all from Sigma-Aldrich Chemical Co., Inc., Milwaukee, WI).

Five plates per each experimental group were fabricated. Polymerization of the resin was carried out in water at 55 ± 1°C in a pressurized chamber (22 psi) for 15 minutes. Each plate was divided into five equal strips producing 25 samples per experimental group. These oversized strips were milled to the digitally calibrated dimensions [10 mm (W)  × 65 mm (L)  × 2.5 mm (D)] and polished to minimize surface roughness. The samples were washed with distilled water to remove any residual monomer and then stored in distilled water at 37°C for 50 ± 2 hours before testing.

### 2.4. Mechanical Testing

Utilizing a 3-point flexural test, the samples were mounted in a calibrated Instron Universal Testing Machine (Instron Corp., Canton, MA). Each plastic strip was supported on each end by metal rollers 50 mm apart. A centrally located rod applied a load until fracture occurred at a uniform crosshead speed of 2.5 mm/min. Force-deflection curves and a complete stress versus strain history for each test were obtained. An Instron computer program was used to calculate the transverse strength, transverse deflection, flexural strength, and modulus of elasticity from the data curves along with the means and standard deviations for each experimental group.

### 2.5. Statistical Analysis

The mean, median, and mode were calculated for each experimental group. Distribution curves were analyzed for normality and One-way Analysis of Variance (ANOVA) and Scheffe Post test were used to compare means between groups.

## 3. Results

**Figure 1:**
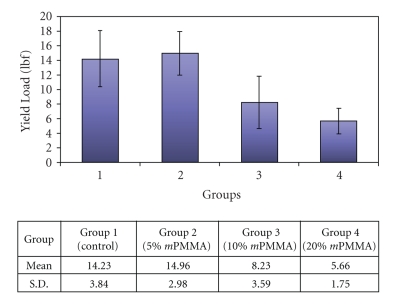
The bar graph represents the mean and standard deviation values for transverse strength or force at fracture for each of the experimental groups.

A representation of the difference in mean transverse strength is shown in Figure 1. The 5% *m*PMMA group showed the highest mean force required to fracture the specimens. A comparison of mean transverse strength revealed no significant difference between the Control and the 5% *m*PMMA group. As the ratio of methacrylic acid:MMA increased, the transverse strength decreased. The 20% *m*PMMA group showed a decrease in transverse strength that was statistically significant compared to the 5% *m*PMMA group (*P* < .05).

**Figure 2:**
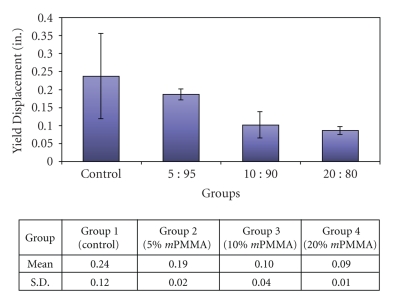
The mean and standard deviation values for transverse deflection for each of the experimental groups.

The transverse deflection measurements and the mean values are shown in Figure 2. The higher the deflection of the specimen was, the farther the crosshead needed to travel to fracture the specimen. In materials with similar transverse strength, the material with higher transverse deflection is more flexible. Results showed that as the ratio of methacrylic acid:MMA increased, the transverse deflection decreased, indicating a decrease in its flexibility. A comparison of mean transverse deflection revealed significant differences between the Control and all groups (*P* < .05) except the 5% *m*PMMA group.

**Figure 3:**
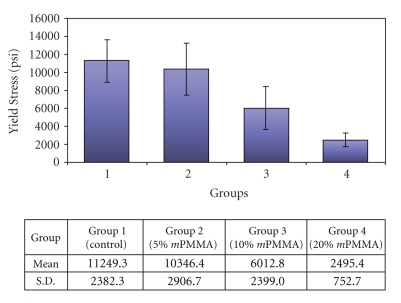
Representation of the mean and standard deviation values for flexural strength for each of the experimental groups.

Figure 3 shows the mean and standard deviation values for flexural strength for each of the experimental groups. The higher the load or force required to fracture the specimens, the higher the fracture resistance. As the ratio of methacrylic acid:MMA increased, the flexural strength decreased. Moreover, the Control showed a significant difference in flexural strength from all other groups (*P* < .05) except the 5% *m*PMMA group.

**Figure 4:**
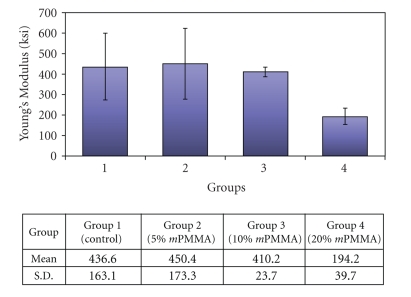
The mean and standard deviation values for Young's modulus of elasticity for each of the experimental groups.

Figure 4 shows the mean and standard deviation values for Young's modulus of elasticity for each of the experimental groups. The elastic modulus is a measure of the stiffness of the material. The higher the elastic modulus is, the more the material will exhibit a lower elastic deformation per unit of stress applied. A comparison between the mean modulus of elasticity of the Control and the 5% *m*PMMA group revealed no significant difference. The 20% *m*PMMA group exhibited the lowest modulus of elasticity, which was significantly lower than both the 5% *m*PMMA group and the commercially available Dental Resin (*P* < .05). Thus, the 20% *m*PMMA group demonstrated the lowest elastic modulus, translating into the least stiff material. The 10% *m*PMMA group did not show any significant difference from the Control or the 5% *m*PMMA group. 

## 4. Discussion

Correlation existed between the physical properties and the anti-fungal activity of surface-charged resins. In the present study, the greatest decrease in transverse and flexural strengths occurred when the ratio of methacrylic acid content was increased from 5% to 10% *m*PMMA (*P* < .05). Interestingly, it was also between these two groups that the most significant reduction in adhesion of *C*. *albicans* occurred [16]. As the ratio of methacrylic acid content was increased, the adhesion of *C. albican* to resin surfaces decreased; however, the physical properties declined in consequence.

The 5% *m*PMMA group was comparable to the Control (Dental Resin) and did not exhibit any significant difference in any parameter tested. The 5% *m*PMMA group produced a higher transverse strength and modulus of elasticity than the Dental Resin; however, it was not statistically significant. This may be attributed to the method of fabrication of the modified resin samples. The experimental resins were not optimized for dental use whereas the Dental Resin has been produced specifically to enhance these physical characteristics. It was notable that the 5% *m*PMMA sample, although not designed for dental use, still had high transverse and flexural strength values that were comparable to the Dental Resin.

In the present study, prepolymerizing or mixing two different types of monomers, methacrylate, and methacrylic acid produced a copolymer. Methacrylic acid is a small molecule with a free carboxyl group providing a negative charge at physiologic pH. Steric interactions can be postulated as the free carboxyl group altering the spatial structure of the new polymer, thereby affecting its physical properties. By creating an ionic molecule, steric hindrance probably causes repelling forces within the resin material. The influence of these internal forces becomes apparent when a material is subjected to physical testing such as compressive and tensile forces. Increasing the methacrylic acid content decreased the flexural and transverse strengths of the resin samples, which probably resulted from an increase in internal repulsive forces. The negative internal forces also affect the modulus of elasticity. The modulus of elasticity represents the basic response of a material to a force. Fundamentally, the elasticity of a substance is related to the existing interatomic forces of the material. The present study indicated that the 20% *m*PMMA group exhibited the lowest modulus of elasticity and had the greatest ionic charge.

 The overall negative charge may also affect the solubility of the material due to water sorption. Previous research showed that an increase in methacrylic acid content correlated with a decreased contact angle measurement, inferring increased hydrophilicity [16]. Umemoto and Kurata [17] have demonstrated that hydrophobic resins decreased water sorption. In that study, they produced copolymers of hydrophobic monomers (norbonyl and phenyl methacrylate) and methyl methacrylate. Increasing the hydrophobic monomers concentration decreased water sorption with no decrease in mechanical properties. These hydrophobic copolymers exhibited higher compressive and bending strength and similar modulus of elasticity compared to PMMA, respectively. The results further support the understanding of the effects of methacrylic acid on the resin's physical properties. As expected, the modification of PMMA with methacrylic acid altered the physical properties of resin.

In the present study a cold-cured method of resin polymerization was utilized, whereas most dentures are made from a heat-cured acrylic form. Studies have shown that there is no difference in surface roughness between the heat- and cold-cured acrylic resins [18, 19]. For the purpose of investigating modified surface characteristics in microbial adhesion, it is reasonable to assume that the present results are equally applicable to both varieties of polymerization methods.

Further modifications may be needed for the modified resins to improve its physical properties while still exhibiting its beneficial antifungal characteristics. A range of methods have been reported for improving the strength of resins through chemical modification of PMMA and through incorporation of fibers, such as carbon, glass, and polyethylene [20–23]. High-impact acrylic is produced from the incorporation of butadiene styrene rubber into the beads during polymerization. Rubber graft copolymers obtained from this process can improve the impact strength of the denture base by as much as 50% [24]. These resins use a monomer that contains little to no cross-linking agent. Normally, crosslinkers are said to provide the craze resistance in a denture base [25]. High-impact acrylics exhibit a craze-inhibiting effect due to the incorporation of rubber. Fiber reinforcement has also been shown to be effective in improving flexural strength of PMMA [26, 27]. Effective fiber reinforcement is dependent on many variables including the fiber type, number, distribution, and orientation. However, concerns about the possible increased adherence of *C. albicans* to fiber-reinforced denture resin bases have been raised. Studies suggest that exposed fibers may increase surface roughness and provide mechanical retention in vivo [28]. Literature suggests that the surface-charged resins can be further modified to increase its physical strength to achieve both biological and mechanical standards. Future research includes continued elucidation of the ideal ratio of methacrylic acid, followed by methods to improve physical properties for clinical applications.

## 5. Conclusion

Surface-charged resins demonstrate to be promising as a biomaterial that can bring about a desired biological response by decreasing Candidal adhesion. The results of the present study suggest that the modification of PMMA with methacrylic acid changes the physical properties of the resin. However, the mechanical properties of 5% *m*PMMA group were comparable to the commercially available Dental Resin. 

## Figures and Tables

**Table 1:** 

CHEMICAL	ACTION	RATIO
PMMA(*m*PMMA) : MMA	POLYMER : MONOMER	3 : 1 by weight
Benzoyl Peroxide	INITIATOR	1% weight of PMMA
2-Hydroxyethyl methacrylate	CROSSLINKER	0.5% volume of MMA
N,N-Dimethylaniline	ACTIVATOR	0.5% volume of MMA
